# Congenital Talipes Equino-Varus

**Published:** 1907-08-31

**Authors:** 


### Points in Surgery.

### CONGENITAL TALIPES EQUINO-VARUS.

### II. TREATMENT.

The objects of the surgeon in treating any case of talipes are to bring the foot into the normal position and to keep it there. Stress is laid upon the second of these points because it is the one which is more difficult of achievement. It is very rare to find in quite young infants that the deformity will not permit of reduction by manipulation; and this is an excellent reason for starting the treatment as soon after birth as possible. Even in those cases in which one or more tenotomies must be performed before the foot can be manipulated into the normal position, there is no reason to delay. Tenotomy may be quite justifiably performed in the first few weeks of extra-uterine life. In the first class of case, in which the deformity can be reduced without tenotomy, manipulation alone, if begun at once and patiently persevered with, will in all probability be sufficient to effect a cure. It is essential in practising the manipulation to bear in mind the double nature of the deformity which has to be overcome—(1) extension of the whole foot at the ankle joint, and (2) inversion of the anterior part of the foot at the mid-tarsal joint. The movements to be practised are naturally those which oppose these abnormal positions; thus the foot must be firmly everted and flexed at the ankle joint. The limb is steadied with one hand, while with the other the foot is firmly moved as far as it will go without causing the infant pain. It should be held in the new position for half a minute before it is released. It is well not to attempt to correct all the deformities in one movement, but to take them one at a time. For example, the foot should be flexed in this manner for five minutes and then everted for another five minutes. These manipulations should be performed at least three times a day. It is generally impossible for the practitioner to spare sufficient time to conduct the manipulations in person daily; but it is easy to instruct the mother, if she be intelligent, or at any rate the nurse, in the treatment by explaining the rationale of it and demonstrating the method at the outset. The movements must be gentle; if they are not, the pain will cause the infant to become fractious and intolerant of further treatment. In addition to this, massage may profitably be applied to the anterior tibial and peroneal groups of muscles to render them strong enough to hold the foot in the corrected position. This treatment should be persisted in for two or three months. If at the end of this time there is a noticeable improvement the amount of manipulation may be gradually reduced until the child begins to walk. If there is little or no improvement from manipulation alone, the effect of some apparatus to retain the foot in position between the manipulations should be tried. For this purpose nothing is so effective as a malleable metal splint. It consists of a foot-piece and leg-piece of thin metal united by a malleable curved bar of copper. The foot-piece and leg-piece are well padded, and when the splint is applied to the limb the exercise of a moderate amount of force will suffice to bend the splint into the required position; the bar of copper is just so rigid that it will successfully resist the efforts of the foot to reassume its deformed position. This splint is not expensive, is easy of application, and will fit one child as well as another. This line of treatment, if patiently persisted in, rarely fails; so that when the child begins to walk the foot is held in the normal position. But the patient must be watched so that the least sign of recurrence of the equino-varus position may be guarded against, and should this happen, treatment must be begun again. If a relapse occurs after the child has begun to walk, it is often sufficient to order a surgical boot which is so constructed that it will not allow inversion of the foot or extension at the ankle.

So far only these cases have been considered in which the deformity can be corrected by manipulation alone. In the second class of case, as already mentioned, complete reduction is rendered quite impossible by the contraction and shortening of the soft parts round the ankle; and here it is necessary to commence treatment with one or more tenotomies. Tenotomy in the neighbourhood of the ankle is done subcutaneously, because there is no risk of damaging important structures, and is quite a simple operation. A small incision is made at the edge of the resisting structure with a sharp tenotome. A blunt tenotome is now inserted by the side of the former, which is withdrawn. It is a matter of small moment whether the tendon is cut away from or towards the surface. In the former method there is more risk of injuring deep structures; the posterior tibial artery or its branches may be cut in dividing the tibialis posticus; but the hæmorrhage so caused is never serious, especially in an infant; and although the writer has seen this accident occur several times, the pressure of a pad and bandage has always been sufficient to arrest the bleeding. On the other hand, in cutting towards the surface it is a surprisingly easy thing to cut or button-hole the skin when the tendon suddenly gives way. The structures which must be divided in talipes equino-varus are the tibialis posticus, the tendo Achillis, and the plantar fascia; occasionally also the tibialis anticus and the flexor longus digitorum. Here again the dual nature of the deformity must be taken into account. Many surgeons divide all resistant structures simultaneously, or divide the tendo Achillis alone. As Mr. Tubby has pointed out, the moment the tendo Achillis is divided, it is almost impossible to correct the varus element completely, because the surgeon no longer has a *point d'appui* from which to apply his force. First, therefore, the structures which are holding the foot in a varus position should be divided, and when that half of the deformity is corrected, the equinus half can be dealt with by tenotomy of the tendo Achillis.

(*To be continued.*)

